# Integrative analyses of immune-related biomarkers and associated mechanisms in coronary heart disease

**DOI:** 10.1186/s12920-022-01375-w

**Published:** 2022-10-20

**Authors:** Lianbo Zhang, Guibin Li, Bo Liang, Xiaoli Su, Haolin Xie, Hongxia Sun, Ge Wu

**Affiliations:** 1 Department of Clinical Pharmacy, Jilin Province FAW General Hospital, Changchun, China; 2 Department of Orthopaedics, Jilin Province FAW General Hospital, Changchun, China; 3 Department of Cardiology, The Second Affiliated Hospital, School of Medicine, Zhejiang University, Hangzhou, China; 4 Department of Human Resources, Jilin Province FAW General Hospital, Changchun, China; 5 Medical Association Office, Jilin Province FAW General Hospital, Changchun, China; 6 Department of Pharmacology, School of Pharmacy, Beihua University, Jilin, China

**Keywords:** Coronary artery disease, Immune, miRNA-mRNA-TF network, SNP-miRNA network

## Abstract

Various studies showed that the effect of immune activation is pro-atherogenic and coronary heart disease (CHD) should therefore be considered an autoimmune disease. This study aimed to identify potential immune-related biomarkers, pathways, and the potential regulatory networks underlying CHD. Differentially expressed genes (DEGs) between CHD and control samples were determined by analyzing GSE71226 and GSE9128. The overlapping differential expression immune-related genes (DE-IRGs) for CHD were identified by analyzing the ImmPort database and two GEO databases. A total of 384 DE-IRGs were identified. Subsequently, comprehensive enrichment analyses suggested that DE-IRGs were enriched in immune-related pathways, including autoimmune thyroid disease, the intestinal immune network for IGA production, and downstream signaling events of B cell receptors. The signature of DE-IRGs was validated using an external independent dataset GSE20681 (AUC = 0.875). Furthermore, we conducted protein–protein interaction network analysis and identified eight hub genes, which were most enriched in regulation of defense response, NF-κB signaling pathway, regulation of JNK cascade, and regulation of cytokine production. Moreover, networks of miRNAs-mRNAs and transcription factors (TFs)-mRNA underlying the integrated data were established, involving eight miRNAs and 76 TF-targeting hub genes. Ultimately, 17 SNPs in miRNA-mediated gene networks were identified. We screened potential immune-related genes in CHD and constructed miRNA-mRNA-TF and SNP-miRNA networks, which not only provide inspired insights into the occurrence and the molecular mechanisms of CHD but also lay a foundation for targeting potential biomarkers using immunotherapy and for understanding the molecular mechanisms of CHD.

## Introduction

Coronary heart disease (CHD) is considered a serious threat to human health worldwide, which caused 365,914 deaths in 2017. Approximately 2 in 10 deaths due to CHD are of adults less than 65 years of age [1]. Millions of patients with chest pain symptoms are commonly admitted to hospitals in high-income countries every year. Approximately 50% of them are diagnosed with CHD, including stable angina, unstable angina, and acute myocardial infarction [2]. In China, the number of CHD patients is 11 million in 2017; the mortality rate was higher in rural residents (122.04/100,000) as compared to that in urban residents (115.32/100,000). The mortality rate in males is higher than that in females, showing an upward trend since 2012 [3, 4].

Comprehensive studies on the mechanism of CHD have recognized that the effect of immune activation is pro-atherogenic, and CHD should therefore be considered a type of autoimmune disease [5–7]. Various cells and molecules involved in the immune response constitute a complex immune mechanism of cooperation and interaction [8–10]. Multiple classic inflammation-related pathways are involved in the survival, proliferation, and migration of immune cells [11–15]. However, there is currently no systematic model to evaluate the immune environment of CHD patients.

CHD was precisely controlled by microRNAs (miRNAs) at the post-transcriptional level and transcription factors (TFs) at the transcriptional level [16, 17]. MiRNAs are a type of endogenous small non-coding RNAs of 20–24 nucleotides in length that are mainly involved in translational repression and/or mRNA destabilization. The widespread availability of high-throughput technologies, including microarray profiling and genome sequencing, has enabled the screening and discovery of an increasing number of differentially expressed (DE) miRNAs that are involved in multiple cardiac transcriptional and signal regulatory pathways [18–20]. TFs are fundamental factors in transcriptional and post-transcriptional regulation, which involves physiological and pathological processes of cells by binding to particular DNA sequences [21]. Although an increasing number of miRNAs and TFs are involved in the immune regulation of CHD, potential miRNA biomarkers and molecular mechanisms remain largely unknown.

**Fig. 1:**
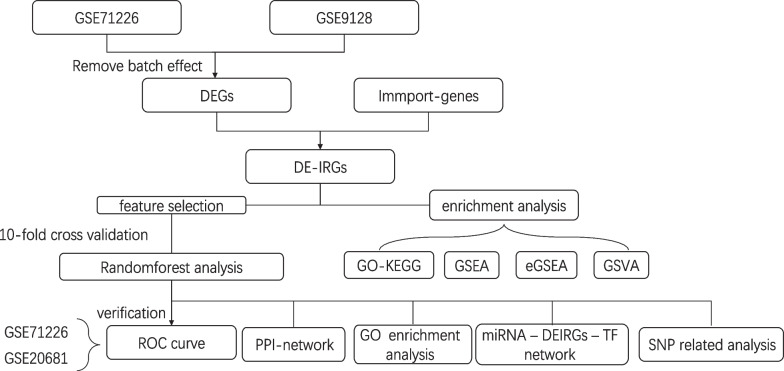
Flow chart of the study

In our study, we screened differential expression immune-related genes (DE-IRGs) for CHD using bioinformatics analysis of multiple microarray databases. Thereafter, we further revealed the hub genes from a protein–protein interaction (PPI) network that was constructed. Furthermore, we constructed miRNA-TF networks to further clarify the crucial immune mechanisms underlying CHD. The newly established immune-related prognostic model was further validated in an independent Gene Expression Omnibus (GEO) database. Ultimately, we analyzed the SNPs that may be involved in the transcriptional regulation of target miRNAs. This study revealed the integrative network of DE-IRGs genetically related to CHD (Fig. 1), developing a theoretical framework applicable to the systems biology for shedding light on the molecular mechanisms underlying CHD. In the future, CHD therapy may turn to specific molecules and cells involved in the immune system, such as monoclonal antibodies against various cytokines and adhesion factors, and gene therapy, to open new ways for the prevention and treatment of CHD.

## Materials and methods

### Microarray data download and preprocessing

All analysis steps are performed using R software (version 4.0.3, https://www.r-project.org/). The gene expression profiles GSE71226 [22], GSE9128 [23], and GSE20681 [24, 25] were retrieved from GEO (https://www.ncbi.nlm.nih.gov/geo/) [26] (Table 1). All samples were from the peripheral blood of *Homo sapiens*. The platform for GSE71226 was GPL570 [HG-U133_Plus_2] Affymetrix Human Genome U133 Plus 2.0 Array, which included three healthy individuals and three CHD patients. The platform for GSE9128 was GPL96 [HG-U133A] Affymetrix Human Genome U133A Array, which collected samples from three healthy individuals and four patients with ischemic cardiomyopathy. The platform for GSE20681 was GPL4133 Agilent-014850 Whole Human Genome Microarray 4 × 44 K G4112F, which contained samples from 4 healthy individuals (CHD prognostic index less than 5) and 36 CHD patients (CHD prognostic index greater than 80) [27]. Series matrix files of GSE71226 and GSE9128 were downloaded to screen and verify differentially expressed genes (DEGs) involved in the coronary subjects.

**Table 1:** Microarray datasets in this study and their experimental designs

Series	Platforms		Tissue source	Design	References
GSE71226	GPL570	Affymetrix Human Genome U133 Plus 2.0 Array	Human sapiens	3 coronary heart disease and 3 healthy people	[22]
GSE9128	GPL96	Affymetrix Human Genome U133A Array	Human sapiens	12 ischemic cardiomyopathy and 12 age- and gender matched controls	[23]
GSE20681	GPL4133	Agilent-014850 Whole Human Genome Microarray 4 × 44 K G4112F	Human sapiens	99 patients with ≥ 50% stenosis in ≥ 1 major vessel by quantitative coronary angiography and 99 controls have luminal stenosis of < 50% by quantitative coronary angiography	[24, 25]

We further analyzed the original files through background correction and quantile normalization via the "normalize between arrays" function in the *limma* package (3.52.2) [28]. The *hclust* package was used for clustering with the default method (“complete”) and “fviz_dend function” in the *factoextra* package was used for visualization. Next, we checked the batch effect by principal component analysis (PCA) through the "remove batch effect" function in the *limma* package. The effect of inter-sample correction was shown by the PCA map using the “fviz_pca_ind” function in the *FactomineR* package [29].

### Identification of differentially expressed immune-related genes (DE-IRGs)

We screened the DEGs from two GEO datasets using the *limma* package, with the threshold criterion of |log_2_FC|> 1 and adj. *p* value < 0.05. The volcano plots and the heatmaps of DEGs were plotted using the *ggplot2* package [30] and the *pheatmap* package (1.0.12) [31], respectively. Thereafter, we collected the data on immune-related genes from the Immunology Database and Analysis Portal (ImmPort Database, https://immport.niaid.nih.gov) [32]. The overlapping DE-IRGs were screened from the GEO dataset and the ImmPort database for further analysis, and the online tool Draw Venn Diagram (http://bioinformatics.psb.ugent.be/webtools/Venn/) was used to display these overlaps.

### Functional and pathway enrichment analysis

Gene ontology (GO) [33] and Kyoto Encyclopedia of Genes and Genomes analysis (KEGG) [34] enrichment analysis of DE-IRGs were conducted using the *clusterProfiler* package (4.4.4) [35]. GO analysis includes the following three criteria: molecular function (MF), cellular component (CC), and biological process (BP). The *enrichplot* package (1.16.1) [36] was used to draw circles, histograms, and bubble diagrams. An adjusted *p* value (from Benjamin-Hochberg method) of less than 0.05 was defined as statistically significant. The data were visualized via the clusterProfiler plug-in (significance was indicated by *p* < 0.05 and *q*-value < 0.05), CluePedia plug-in [37], and ClueGO plug-in [38] in Cytoscape software (http://www.cytoscape.org/) (kappa score ≥ 0.4).

### Gene set enrichment analysis (GSEA) and xPierGSEA

The biological pathways that were significantly altered in CHD samples as compared to the control samples were determined using GSEA. GSEA [39] was performed using *cluster profiler* package, with c2.all.v7.4.symbols.gmt as the reference gene set, and analyzed using the GSEA function from the GSEA database and the gseaplot2 function of the *enrichplot* package. False discovery rate (FDR) < 0.25 and *p* value < 0.05 were set as the cut-off criteria. xPierGSEA (2.9.0) was implemented by the *Pi* package [40], providing us with other alternatives to screen significantly differential biological functions in CHD.

### Gene set variation analysis (GSVA)

GSVA [41] was carried out to explore the expression matrix of DE-IRGs using the *GSVA* package, which does not need to group samples in advance and calculates the enrichment score of the specific gene set in each sample. C2: curated gene sets were selected as the reference gene set, with the t value of GSVA scores 0.5 as the cut-off value standard.

### Construction of gene feature selection and random forest (RF) diagnosis model

To improve the performance of the diagnosis model, specific variable genes related to the classification of CHD outcomes were screened by the Boruta feature selection method (18). The RF diagnostic model classifier was implemented using a *random forest* package (1.18.1) [42], which distinguished patients with and without CHD in the combined data set (GSE71226, GSE1128). DE-IRGs were selected as disease-associated genes for follow-up model building. Next, the average mistranscription rates of all genes were calculated according to the out-of-bag data. The best variable (mtry) of binary trees in the node was set to 2, and 1000 was selected as the best number of trees in the RF. Thereafter, the RF model was built and the importance value was obtained by employing the Gini coefficient method. The accuracy of the RF model was tested by using the tenfold cross model, and the accuracy of the model tended to be stable when the number of genes was confirmed. The *pROC* package (1.17.0) [43] was used to validate AUC classification performance. External validation of the immune-related signature was conducted using an external dataset (GSE20681).

### Construction of PPI network and identification of hub genes

To further study the biological function of DE-IRGs, we constructed a PPI network (https://string-db.org/) [44, 45] and cell landscape (https://metascape.org/) [46] with a combined score ≥ 0.4. Moreover, we used the Cytoscape software [47, 48] to visualize the PPI network. We employed the Molecular Complex Detection (MCODE) [49], a plug-in of Cytoscape, to identify vital modules (MCODE score ≥ 4). We applied the CytoHubba [50], another plug-in of Cytoscape, to indicate essential nodes in the network and hub genes involved in the PPI network.

### Construction of a miRNA-DE-IRGs-TF regulatory network

We used NetworkAnalyst (https://www.networkanalyst.ca/) [51] to analyze miRNA databases and predict the interaction between miRNA and DE-IRGs using TargetScan [52] and miRDB [53, 54]. Predicted_Targets_ predictions.txt file was downloaded from the TargetScan official website, and miRDB_v6.0_prediction_result.txt was collected from miRDB. Furthermore, we predicted the interaction of DE-IRGs and transcription factors via the TRRUST database (https://www.grnpedia.org/trrust/) [55]. Ultimately, we visualized miRNA-gene and TF-gene networks using Cytoscape software.

### Predictive analysis of SNP affecting miRNA binding to DE-IRGs

We used the miRNASNP database [56] to predict the SNPs in the seed region of the potential candidate miRNAs. We identified genes affected by SNP mutations in the seed region of potential candidate miRNAs and then selected the DE-IRGs to form a list of miRNA-SNP-DE-IRGs combinations. Finally, we constructed the miRNA-SNP-DE-IRGs interaction network according to the list. If miRNA was not completely labeled 3p/5p or two highly homologous miRNAs appeared, both of them were searched.

## Results

### Identification of DEGs in CHD

**Fig. 2:**
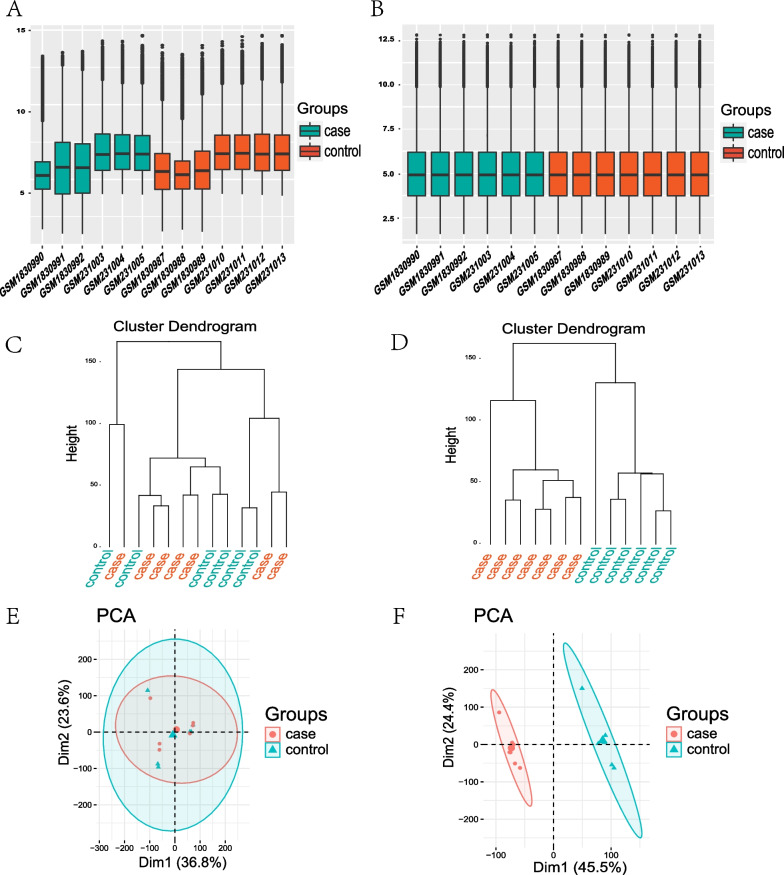
Preprocessing of GSE71226 and GSE9128. **A** and **B** Box plots of DEGs before and after standardization of two datasets. **C** and **D** Cluster dendrogram of DEGs before and after batch correction. **E** and **F** PCA plots of datasets before and after batch correction

**Fig. 3:**
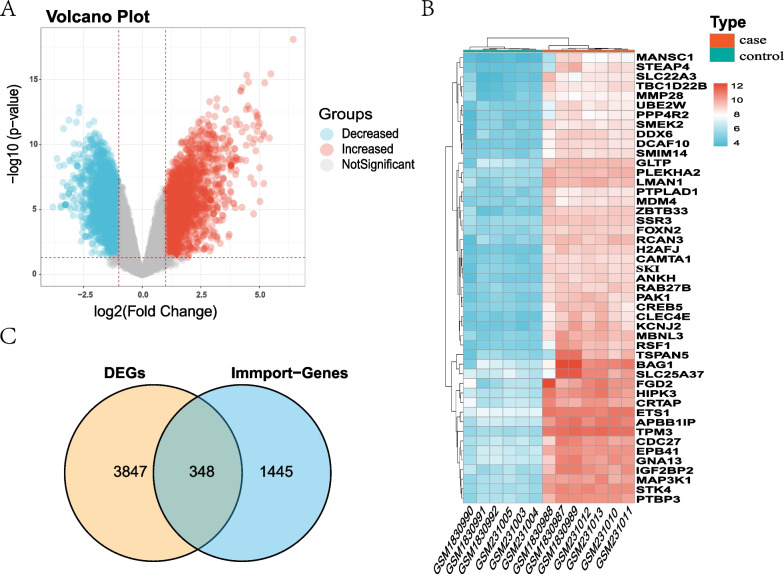
Identification of DEGs and DE-IRGs. **A** Volcano plots of DEGs. 2070 red points represent upregulated genes, 2126 blue points indicate downregulated genes, and 10,002 grey points represent unchanged genes. **B** Heatmaps of DEGs with |log2FC|> 4 and adj. *p* value < 0.001. Blue represents the case group and red represents normal control. **C** Venn Diagram demonstrates the intersection of DEGs and immune-related genes

The gene expression profiles of GSE71226 and GSE9128 datasets were merged, and standardized, and the batch effects were removed. Gene expression profiles before and after standardization are displayed in box plots (Fig. 2A, B). Gene expression profiles before and after batch correction are displayed by Cluster dendrogram (Fig. 2C, D) and PCA (Fig. 2E, F), which showed that the control group and CHD group were clustered into two groups after batch correction, indicating the heterogeneity of sample sources. After data were pre-processed, the volcano plot indicated that 4196 DEGs were extracted from two gene expression profiles with 2070 DEGs being up-regulated and 2126 DEGs being down-regulated (Fig. 3A). Then we chose these DEGs with |log_2_FC|> 4 and adj. *p* value < 0.001 for visualization in the heatmap (Fig. 3B).

### GO and KEGG enrichment analyses

**Fig. 4:**
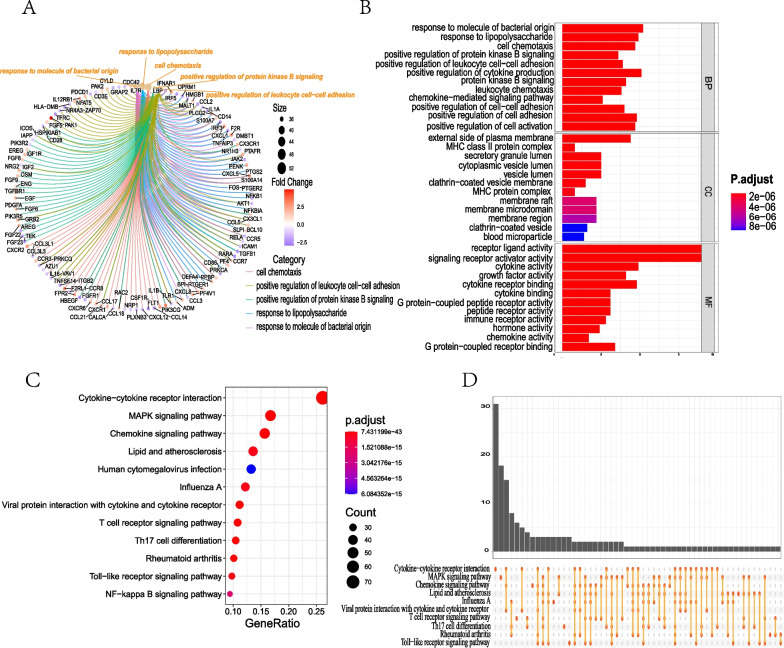
Functional enrichment analyses of DE-IRGs. **A** GO enrichment analysis with “eGO” function (OrgDb = org.Hs.eg.db, pvalueCutoff = 0.05, qvalueCutoff = 0.25, pAdjustMethod = "BH") in the *clusterProfiler* package. The results were visualized with the “cnetplot” function in the *enrichplot* package and the color of the dot marks the logFC of the gene. **B** GO enrichment analysis. The top 12 results were visualized with the “barplot” function in the *enrichplot* package. The x-axis and y-axis represent the gene ratio, and GO terms, respectively, and the color represents the corrected *p* value. **C** KEGG pathway enrichment analysis with “enrichKEGG” function (organism = "hsa", keyType = "kegg", pvalueCutoff = 0.05, pAdjustMethod = "fdr") in the *clusterProfiler* package. The top 12 results were visualized with the “dotplot” function in the *enrichplot* package and the x-axis and y-axis represent the gene ratio and pathway, respectively. **D** Correlation between KEGG enrichment pathways with the “upsetplot” function in the *enrichplot* package and the line represents the correlation between pathways

A total of 348 overlapping DE-IRGs (Fig. 3C), the intersection of DEGs and ImmPort genes, integrated by the Venn Diagram online tool were identified (Fig. 3C). GO term and KEGG enrichment analyses were performed on DE-IRGs. The significantly top five GO terms were positive regulation of cytokine production, positive regulation of leucocyte cell−cell adhesion, external side of the plasma membrane, MHC class II protein complex, and immune receptor activity. For BP, DE-IRGs were significantly enriched in response to molecule of bacterial origin, response to lipopolysaccharide, cell chemotaxis, positive regulation of protein kinase B signaling, positive regulation of cytokine production, and positive regulation of leukocyte cell–cell adhesion. For CC, DE-IRGs were significantly enriched in the external side of the plasma membrane, MHC class II protein complex, secretory granule lumen, cytoplasmic vesicle lumen, and vesicle lumen. For MF, DE-IRGs were significantly enriched in receptor-ligand activity, signaling receptor activator activity, cytokine activity, growth factor activity, and cytokine receptor binding (Fig. 4A, B). The top five significant enrichment pathways indicated by KEGG analysis were Cytokine-cytokine receptor interaction, Chemokine signaling pathway, Viral protein interaction with cytokine and cytokine receptor, T cell receptor signaling pathway, and Rheumatoid arthritis (Fig. 4C, D).

### GSEA, xPierGSEA, and GSVA

**Fig. 5:**
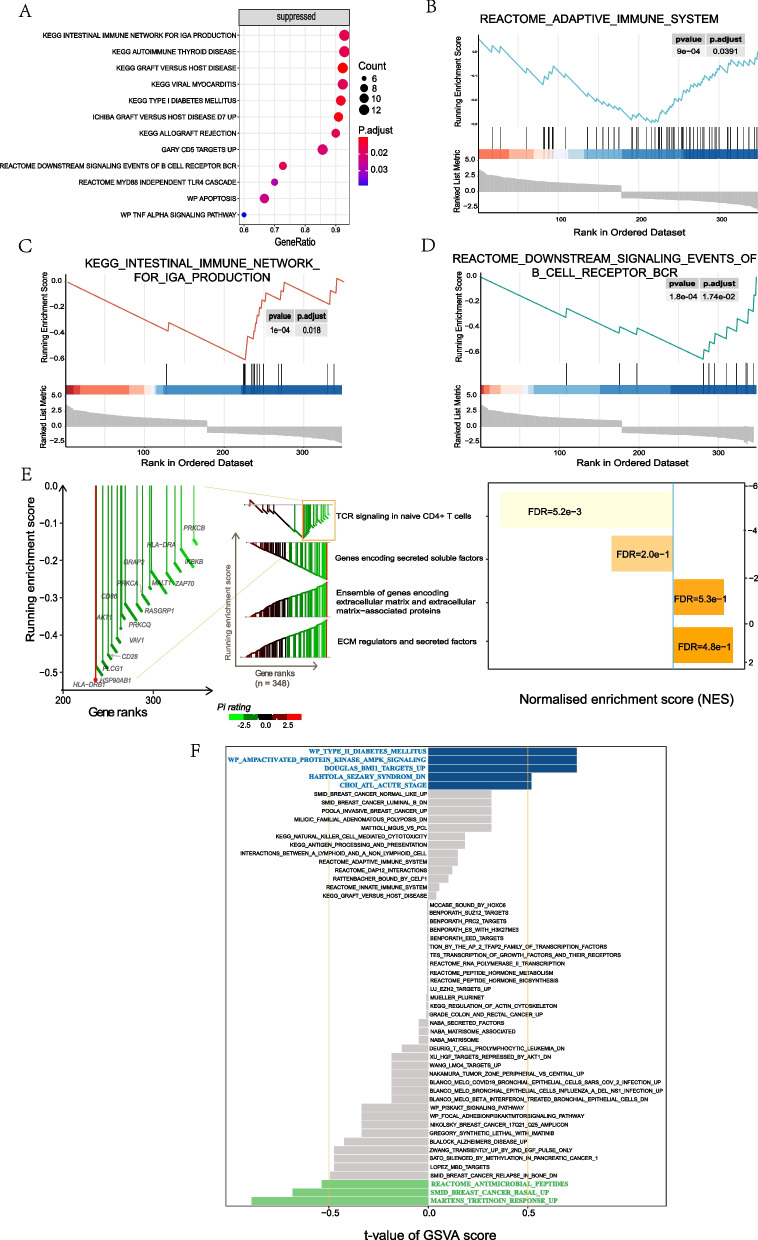
Enrichment pathway analysis. **A** GSEA plots. The x-axis represents the proportion of DEGs, the color represents the corrected *p *value (corrected by Benjamin-Hochberg test), and the dot represents the number of enriched genes. **B**–**D** Predictive immune-related pathways in CHD via GSEA. **B** Autoimmune thyroid disease. **C** Intestinal immune network for IgA production. **D** Downstream signaling events of B cell receptor. **E** xPierGSEA of the top four significant pathways. The leading edge on the left chart reveals leading targets and the bar chart on the right shows the FDR value (numerical value) and NES value. **F** GSVA (blue: T-score > 0.5, green: T-score < 0.5, gray: not significant)

GSEA indicated that the most significantly enriched gene sets included the intestinal immune network for IgA production and KEGG autoimmune thyroid disease (Fig. 5A–D). The xPierGSEA showed that DE-IRGs play a significant role in the immune mechanism of CHD, especially in the T-cell receptor (TCR) signaling pathway of Na ï CD4 + T cells (FDR = 5.2e−3) (Fig. 5E). GSVA indicated that the same cellular mechanism may occur in multiple diseases other than CHD, such as type II diabetes mellitus, breast cancer basal up, etc. (Fig. 5F).

### Diagnosis model construction and analysis of DE-IRGs

**Fig. 6:**
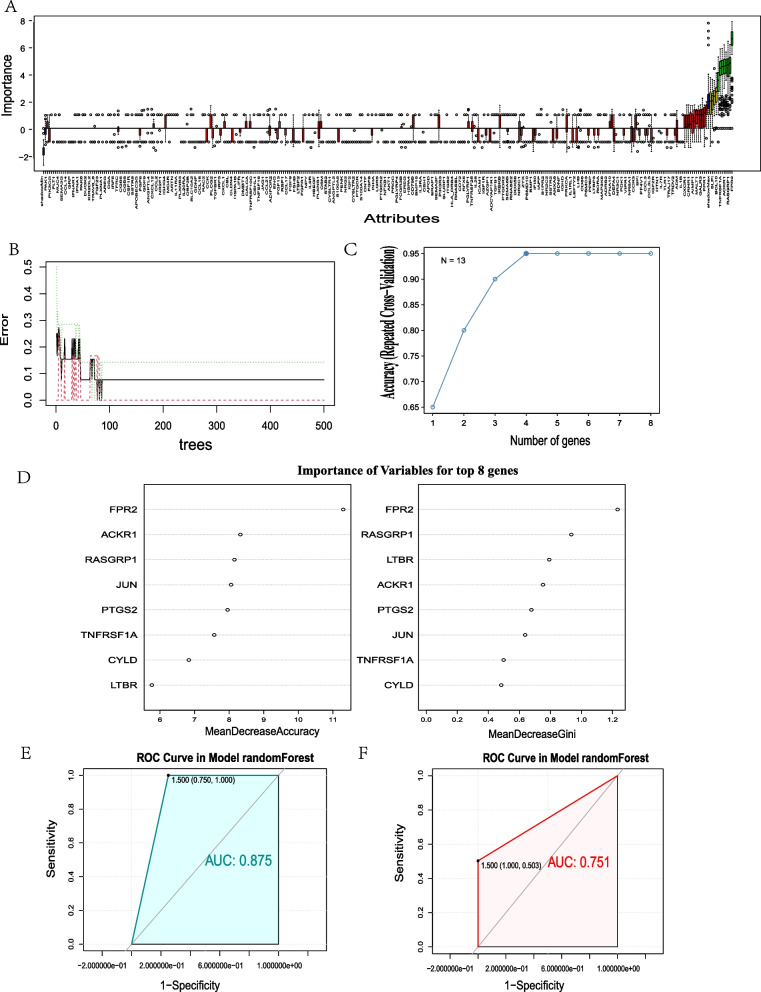
Hub gene screening, diagnostic model construction, and validation of DE-IRGs. **A** Box graph of random forest selection. The green marker is an important characteristic gene. **B** Error rate under the random forest diagnosis model gradually decreases with the increase of ntree, and finally tends to be stable. **C** The accuracy under tenfold crossover model tends to be stable (> 0.95) when the fourth gene is included. D. Random forest assessment of eight hub genes. *FPR2* displays were much more important than other genes. **E** ROC curve and an area under ROC (AUC) value of GSE71226 data set (AUC = 0.875). **F** ROC-AUC value of GSE20681 data set (AUC = 0.751)

**Fig. 7:**
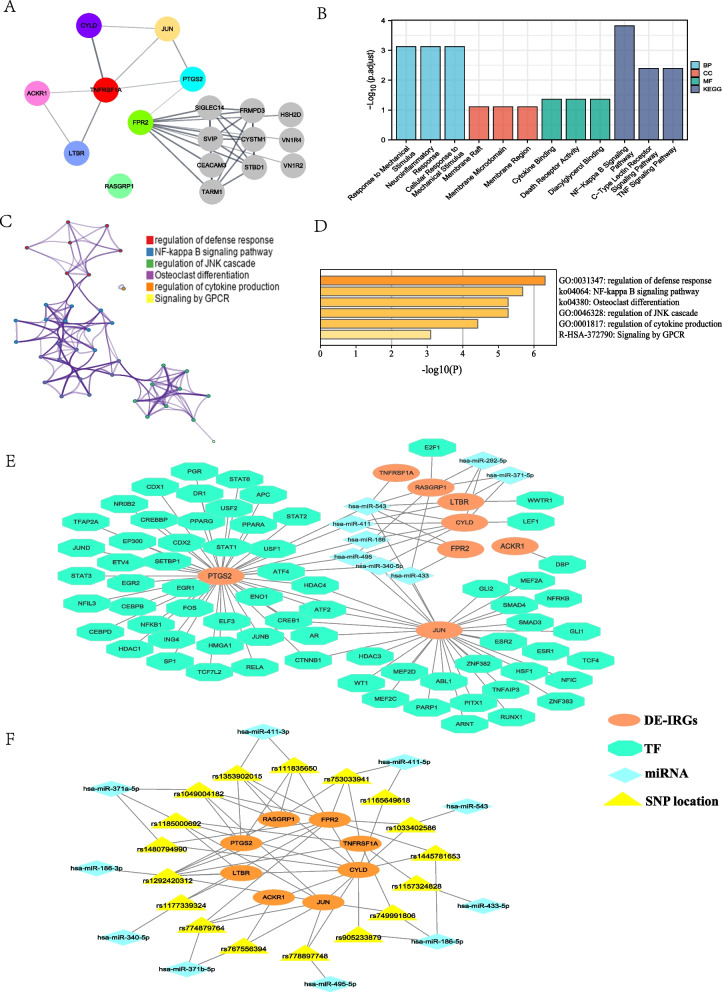
Functional enrichment analysis of hub genes and miRNA-DE-IRGs-TF regulatory network. **A** PPI network of DE-IRGs. **B** Cluster profiler showed that the main functions were enriched in NF-kappa B signaling pathway and TNF signaling pathway. **C** Metascape network diagram, Metascape functional enrichment correlation analysis of hub gene. **D** Metascape histogram. The first six functions with the lowest p value were selected. **E** miRNA-DE-IRGs-TF regulatory network. **F** miRNA-SNP-DE-IRGs network

Eight hub genes (LTBR, ACKR1, FPR2, PTGS2, JUN, RASGRP1, CYLD, and TNFRSF1A) from the DE-IRGs were screened by Boruta algorithms feature selection (Fig. 6A). The Metascape online analysis demonstrated that the main functions were predominantly enriched in regulation of defense response, NF-kappa B signaling pathway, regulation of JNK cascade, and regulation of cytokine production (Fig. 7C, D). The cluster profiler showed that the main functions were enriched in the NF kappa B signaling pathway and TNF signaling pathway (Fig. 7B).

In the construction of the random forest diagnosis model, eight hub genes of the Boruta feature selection were recruited to establish the signature (Fig. 6B). The tenfold model verified the accuracy of the random forest model. The accuracy increased concordantly with the growing proportion of gene features. Our model accuracy tended to be stable when the fourth gene was included (> 0.95) (Fig. 6C). According to the results of Mean Decrease Gini, the eight characteristic genes were ranked in decreasing order of importance as *FPR2*, *RASGRP1*, *LTBR*, *ACKR1*, *PTGS2*, *JUN*, *TNFRSF1A*, and *CYLD* (Fig. 6D). AUC was used to evaluate the random forest model. The AUC value of GSE71226 is 0.875 (Fig. 6E) and the AUC value of external validation set GSE20681 is 0.751 (Fig. 6F), indicating that the model has high efficiency in predicting the diagnosis and classification of CHD.

### PPI network construction and hub gene selection

The PPI network included 18 nodes and 28 edges. The eight colored nodes represented hub genes, and the gray nodes are extended genes with matching confidence cut-off (cut-off score is 0.4) by the String app expand network (Fig. 7A). The results show that *FPR2* is at the center of the hub gene and extended gene, revealing that it not only serves as a bridge to communicate with other functional networks but also plays a more important biological role than other key genes in the network.

### Construction of miRNA-DE-IRGs-TF network

Based on two miRNA target gene databases (TargetScan and miRcode), eight miRNAs were obtained, which were hsa-miR-411, hsa-miR-371-5p, hsa-miR-292-5p, hsa-miR-186, hsa-miR-495, hsa-miR-543, hsa-miR-340-5p, and hsa-miR-433. TRRUST transcription factor database predicted the targeted DEGs for TFs. Overall, 76 pairs of TF-DE-IRGs interaction groups were found. Among them, *PTGS2* and *JUN* predicted the most TFs, and the STAT family TFs (STAT1, STAT2, STAT3, and STAT6) matched *PTGS2* the most (Fig. 7E). *FPR2* and *TNFRSF1A* did not predict matching TFs.

### Predictive analysis of SNP-influenced miRNAs and DE-IRGs

Potential candidate miRNAs targeting DE-IRGs were analyzed using miRNASNP to determine how the SNP variants in the seed region of the miRNAs affected their binding to the 3' UTR region of DE-IRGs, and to construct miRNA-SNP-DE-IRGs interaction networks (Fig. 7F). A total of 17 SNP locations were identified (rs1165649618, rs753033941, rs111835650, rs1353902015, rs1185000692, rs1049004182, rs1480794990, rs774879764, rs767556394, rs1292420312, rs749991806, rs1445781653, rs905233879, rs778897748, rs1033402586, rs1177339324, and rs1157324828), which may play regulatory roles in the binding of miRNAs to DE-IRGs. Among them, 13 SNP mutations increased the number of miRNAs that could target the 3'-UTR of the DE-IRGs (gain), whereas 11 SNP mutations had the opposite effect (loss) (Table 2).

**Table 2:** SNPs predicted to potentially affect miRNA binding

Target_Gene	SNP location	miRNA (loss)	miRNA (gain)
ACKR1	rs767556394		hsa-miR-371b-5p
CYLD	rs1165649618	hsa-miR-411-5p	
	rs111835650	hsa-miR-411-3p	
	rs1353902015	hsa-miR-411-3p	
	rs1185000692	hsa-miR-371a-5p	
	rs1480794990	hsa-miR-371a-5p	
	rs774879764	hsa-miR-371b-5p	
	rs1292420312		hsa-miR-186-3p
	rs1445781653	hsa-miR-186-5p	
	rs905233879	hsa-miR-186-5p	
	rs778897748		hsa-miR-495-5p
FPR2	rs753033941	hsa-miR-411-5p	
	rs1353902015	hsa-miR-411-3p	
	rs1049004182	hsa-miR-371a-5p	
	rs1480794990	hsa-miR-371a-5p	
	rs774879764	hsa-miR-371b-5p	
	rs1292420312	hsa-miR-186-3p	
	rs1445781653	hsa-miR-186-5p	
	rs1033402586	hsa-miR-543	
	rs1177339324	hsa-miR-340-5p	
JUN	rs1185000692		hsa-miR-371a-5p
	rs1049004182		hsa-miR-371a-5p
	rs774879764		hsa-miR-371b-5p
	rs767556394		hsa-miR-371b-5p
	rs1292420312	hsa-miR-186-3p	
	rs749991806		hsa-miR-186-5p
	rs778897748		hsa-miR-495-5p
	rs1033402586	hsa-miR-543	
LTBR	rs1185000692		hsa-miR-371a-5p
	rs1292420312	hsa-miR-186-3p	
	rs1177339324	hsa-miR-340-5p	
PTGS2	rs111835650		hsa-miR-411-3p
	rs1353902015		hsa-miR-411-3p
	rs1049004182	hsa-miR-371a-5p	
	rs1292420312	hsa-miR-186-3p	
RASGRP1	rs753033941	hsa-miR-411-5p	
	rs111835650	hsa-miR-411-3p	
	rs1353902015	hsa-miR-411-3p	
	rs1049004182	hsa-miR-371a-5p	
	rs1292420312	hsa-miR-186-3p	
TNFRSF1A	rs753033941		hsa-miR-411-5p
	rs1185000692		hsa-miR-371a-5p
	rs1292420312	hsa-miR-186-3p	
	rs749991806		hsa-miR-186-5p
	rs1157324828		hsa-miR-433-5p

## Discussion

A growing body of evidence has demonstrated that immune-related genes (IRGs) play significant roles in CHD. Various cellular and molecular-mediated immune mechanisms participate in the occurrence of CHD and promote the development and deterioration of CHD [57, 58]. Although it has been investigated widely, the molecular mechanism and critical regulators involved in IRGs for evaluating CHD patients remain to be further elucidated, which will help to explore the complex pathological process of the formation and development of CHD. In this study, 348 DE-IRGs were identified as significant with respect to CHD by integration analysis of two mRNA microarray datasets and ImmPort database. Next, the intersection of GO term and KEGG pathways enrichment analysis indicated that immune-related terms and pathways were highly enriched. The results suggested that immune-related molecular functions and crucial biological processes are involved in CHD pathogenesis. Also, GSEA enriched pathways are mainly involved in the internal immune network for IgA production and autoimmune theroid disease pathways. The xPierGSEA also showed that DE-IRGs played a vital role in the immune mechanism of CHD, being especially involved in the TCR signaling pathway of naïve CD4 + T cells.

Based on the 348 DE-IRGs, we built a PPI network and obtained eight hub genes using MCODE analysis, including *FPR2, RASGRP1, LTBR, ACKR1, PTGS2, JUN, TNFRSF1A*, and *CYLD* to explore the regulatory networks and crucial pathways underlying CHD. Our results demonstrated that *FPR2* is at the center of the network including characteristic genes and extended genes, indicating that it might play a potential role in immunoregulation processes. *FPR2*, a seven-transmembrane G protein-coupled receptor, plays multifaceted functions, such as a role in sensing bacteria and modulation of immune responses [59], colonic epithelial homeostasis, inflammation, and tumorigenesis [60]. *PTGS2* was confirmed to suppress cell viability [61] and proliferation [62] of cells by activating the AKT/NF-κB pathway [63]. Overexpression of *TNFRSF1A* promoted the activation of the NF-κB signaling pathway and plays a potential regulatory role in neuronal cell damage [64]. The variant in *TNFRSF1A* is involved in Tumor Necrosis Factor Receptor-Associated Periodic Syndrome (TRAPS), and can therefore be used as a novel pathogenic therapeutic target [65]. CYLD is a tumor suppressor protein. Studies showed that the loss of function of CYLD can release the polyubiquitin chain in the ubiquitinated substrate, which can then inhibit the IκBα Kinase complex and then inhibit the activation of the NF-κB signaling pathway, which is a negative regulator of this signaling pathway [66]. The relationship between the abovementioned hub genes and the immune-related molecular mechanism of CHD has not been confirmed before. In this study, several hub genes were found to be involved in the activation or inhibition of the NF-κB and TNF pathways. These findings are consistent with the Metascape analysis enrichment result, which illustrated that the eight hub genes were mainly enriched in the regulation of defense response, NF-κB signaling pathway, regulation of JNK cascade, and regulation of cytokine production. As a key and strictly regulated regulator, the NF-κB family of transcription factors is responsible for immune development, immune responses, and inflammation. The false regulation of NF-κB has been implicated in a wide range of diseases ranging from inflammatory to immune disorders [67]. TNF-α, mainly secreted by macrophages and monocytes, can directly damage vascular endothelial cells, cause a pathological inflammatory reaction, reduce the stability of atherosclerotic plaque, lead to the rupture of atherosclerotic plaque, and indirectly and directly accelerate the formation and development of atherosclerotic plaque [68].

CHD is a complicated disease involving various signaling pathways, protein-coding genes, numerous transcription factors, and post-transcriptional regulation of heart morphogenesis, cardiac development, and cardiac muscle cell differentiation and contractility. It is well known that miRNAs control gene expression mostly at the post-transcriptional level, whereas TFs are involved in the activation or inhibition of transcription at a pre-transcriptional level. Studies have implicated the abnormalities in miRNA expression in CHD [69]: miR-34a protects endothelial function in CHD patients [70]; miR-23a improves restoration of blood flow in the ischemic region [71]; miR-135b and miR-499a induce the proliferation of endothelial cells and vascular smooth muscle cell [72]; miR502 suppresses autophagy [73]; miR-33/33b lowers low-density lipoproteins and raises high-density lipoproteins concentrations [74]; miR-939 in angiogenesis [75]; miR-155 prevents the occurrence and development of atherosclerosis and CHD [76]; miR-126 substantially prevents atherosclerotic cardiovascular diseases [77]; miR-21 prevents the formation of neointimal and restenosis of arteries after angioplasty [78]; miR-146 enhances the collateral coronary circulation in ischemic tissue [79]. In this study, we constructed miRNA-TF co-regulatory networks based on candidate immune-related miRNAs, TFs, and hub genes, and performed network analysis to obtain consensus hub nodes, modules, and motifs involved in CHD. Based on two miRNA target gene databases (TargetScan and miRcode), eight miRNAs were obtained, which were hsa-miR-411, hsa-miR-371-5p, hsa-miR-292-5p, hsa-miR-186, hsa-miR-495, hsa-miR-543, hsa-miR-340-5p, and hsa-miR-433, and they were all target genes. Through the prediction of the trust transcription factor database, 76 pairs of TF-DE-IRGs interaction groups were found. *PTGS2* and *Jun* predicted the most transcription factors, and STAT family transcription factors (STAT1, STAT2, STAT3, and STAT6) matched *PTGS2* best. Cluster analysis of the miRNA-DE-IRGs-TF regulatory network revealed that its main functions were promotion or inhibition of the NF-κB signaling pathway and TNF signaling pathway. Among eight miRNAs, miRNA-292-5p [80, 81], which participates in myocardial ischemia–reperfusion injury by activating the peroxisome proliferator-activated receptor-α/-γ (PPARα/PPARγ)-dependent signaling pathway, has been studied since 2018. Recently, several studies have provided evidence that cardiomyocyte-enriched miR-186 contributes to cardiac injury and myocardial infarction as well as the progression of atherosclerosis [82]. The remaining six miRNAs were not retrieved for in-depth study.

Recent studies revealed that miRNA-related Single Nucleotide Polymorphisms (SNPs) might affect disease susceptibility and phenotypes in an SNP-genotype-dependent manner by modifying miRNA regulation [83, 84]. In our study, according to the function enrichment prediction of characteristic genes in Metascape, 17 SNPs were identified. They may play a key role in the binding of miRNAs and DE-IRGs. Most detected SNPs have not been studied yet. Therefore, we recommend combining miRNA expression profiling analysis with genome-wide SNPs to identify potential disease biomarkers for future research.

However, this study has several limitations. Firstly, a comprehensive elucidation of the molecular mechanisms should be conducted to understand the factors underlying the occurrence and development of CHD. Individual samples with varying degrees of CHD need to be collected and studied. Secondly, the low number of patients analyzed in this study likely introduced bias. Larger sample size and comprehensive analysis of the interactions between environmental and genetic factors may better reveal the roles of hub genes and pathways in CHD pathogenesis. Finally, further bioinformatics analyses and validation assays are required for elucidating the gene function and biological mechanisms of the predicted genes in CHD.

Tremendous progress has been made in understanding molecular mechanisms of CHD in vitro, ex vivo, and in vivo. However, we should deepen our understanding of CHD pathogenesis and undertake further investigation to find more feasible treatments. The complex regulatory network involving hub genes, miRNAs, and TFs will be an exciting new field to explore and will shed new light on CHD development. We aimed to discover novel markers that would be helpful in diagnosis, prognosis, prediction, and immune microenvironment detection in CHD patients, and have been successful in finding some very promising leads. Further research in this area will help explore the formation and development of CHD.

## Data Availability

All data generated or analyzed during this study are included in this published article and its supplementary information files. The datasets generated and/or analysed during the current study are available in the GEO repository, https://www.ncbi.nlm.nih.gov/geo/.
